# Exploring the Impact of DNA Methylation on Gene Expression in CRC: A Computational Approach for Identifying Epigenetically Regulated Genes in Multi-Omic Datasets

**DOI:** 10.3390/cancers18020211

**Published:** 2026-01-09

**Authors:** Andrei Stefan Blindu, Silvia Berardelli, Federica De Paoli, Federico Manai, Rossella Tricarico, Susanna Zucca, Paolo Magni

**Affiliations:** 1 Department of Electrical, Computer and Biomedical Engineering, University of Pavia, Via Ferrata, 5, 27100 Pavia, Italypaolo.magni@unipv.it (P.M.); 2 enGenome s.r.l., Via Ferrata, 5, 27100 Pavia, Italy; 3 Department of Biology and Biotechnology “L. Spallanzani”, University of Pavia, 27100 Pavia, Italy; 4 Laboratory of Epigenetics, Istituti Clinici Scientifici Maugeri IRCCS, 27100 Pavia, Italy

**Keywords:** DNA methylation, epigenetic regulation, colorectal cancer, CpG island methylator phenotype, methylation-associated genes, gene expression, computational analysis, multi-omics integration, biomarker discovery

## Abstract

Background/Objectives: DNA methylation is a key epigenetic process that regulates gene expression and is often disrupted in colorectal cancer (CRC). Aberrant methylation of promoter CpG islands can silence tumor suppressor genes and drive tumorigenesis. A subset of CRCs exhibits the CpG Island Methylator Phenotype (CIMP), characterized by widespread hypermethylation and distinct clinical outcomes. Identifying genes whose expression is epigenetically regulated by methylation is important for prioritizing candidate biomarkers and therapeutic targets in CRC. Methods: We developed and compared a series of computational approaches to identify genes whose expression is regulated by DNA methylation in The Cancer Genome Atlas (TCGA) cohort of Colon Adenocarcinoma (COAD) patients. Samples were stratified according to their CpG Island Methylator Phenotype (CIMP) level to capture distinct epigenetic subgroups. The proposed framework integrates methylation and transcriptomic data to systematically detect methylation–expression associations indicative of epigenetic regulation. Results: The best-performing method identified gene sets strongly associated with promoter methylation–expression relationships and enriched for pathways relevant to colorectal cancer progression and patient stratification. To evaluate the robustness and transferability of the approach, it was further validated on independent datasets, including Stomach Adenocarcinoma (STAD), Glioblastoma Multiforme (GBM), and Mesothelioma (MESO), supporting its robustness and potential generalizability across multiple tumor types. Conclusions: Our study highlights the potential of computational pipelines to uncover epigenetically regulated genes in colorectal cancer. The identified candidate genes provide a hypothesis-generating foundation for refining molecular stratification and guiding future studies aimed at epigenetic biomarker discovery and therapeutic hypothesis development.

## Simple Summary

DNA methylation, a process that controls how genes are turned on or off, can become disrupted in colorectal cancer and contribute to tumor development. Some tumors show widespread methylation changes that may silence key genes involved in cell regulation. This study aims to systematically identify genes whose activity is influenced by DNA methylation by integrating large-scale molecular data from colorectal cancer patients. Different computational methods were compared to determine the most effective approach for detecting methylation-related changes in gene expression, and the results were validated in other cancer types. The findings establish a promoter-centric, regression-based framework that prioritizes candidate genes whose expression variability is strongly explained by promoter methylation across CIMP-stratified tumors, which could: (i) refine molecular subclassification of patients beyond traditional CIMP status, (ii) identify candidate diagnostic or prognostic methylation-based biomarkers, and (iii) prioritize genes for functional validation in epigenetic therapy studies.

## 1. Introduction

Analyzing gene expression is critical for elucidating complex biological processes and the mechanisms controlling gene activity under pathological conditions. The aim of this study is to develop, systematically compare, and validate computational strategies for identifying genes regulated by DNA methylation, with an emphasis on interpretability and cross-tumor robustness.

In cancer, key cellular pathways are frequently disrupted due to the abnormal regulation of critical genes, including the silencing of tumor suppressor genes or the activation of proto-oncogenes [1,2]. This alteration of gene expression can be driven by many biological mechanisms, with epigenetic regulations, such as DNA methylation, playing a crucial role [1,2]. DNA methylation is a modification that mainly occurs at cytosine residues in CpG dinucleotides when a methyl group (CH_3_) binds to the cytosine nucleotide. This process is tightly regulated by specific enzymes, including DNA methyltransferases (DNMTs), which catalyze de novo methylation (DNMT3A and DNMT3B) or maintain methylation patterns during DNA replication (DNMT1). Conversely, Ten-Eleven Translocation (TET) enzymes (TET1, TET2, and TET3) catalyze the oxidation of 5-methylcytosine, facilitating active DNA demethylation [3].

Aberrant promoter hypermethylation is generally associated with transcriptional silencing [1,2,4]. Consequently, changes in the methylation status of key genes can significantly impact disease development, progression, and prognosis.

Investigating the interplay between DNA methylation and gene expression is essential for uncovering novel epigenetic biomarkers and understanding tumor-specific regulatory mechanisms. In particular, a subset of tumors exhibit the CpG Island Methylator Phenotype (CIMP) [5], a molecular subtype characterized by widespread hypermethylation of CpG islands in gene promoter regions, leading to gene silencing. Cancer patients can be stratified into CIMP high (CIMP-H), CIMP low (CIMP-L) and Non-CIMP subtypes based on their level of CpG island methylation. Stratifying patients based on CIMP status delineates distinct molecular subtypes with characteristic methylation and genomic features, thereby informing tumor biology, therapeutic stratification, and prognostic assessment [6]. CIMP has been widely studied in Colorectal Cancer (CRC), where it defines distinct molecular subgroups associated with clinical outcomes and therapeutic response [6], and also in other malignancies, including gastrointestinal [7] and brain tumors [8]. In Colorectal Cancer, CIMP-positive tumors have been reported to exhibit distinct molecular features, including frequent BRAF mutations, microsatellite instability, and widespread promoter CpG island hypermethylation, which may contribute to their pathogenesis [6,9,10]. DNA methylation changes have also been explored as diagnostic and prognostic biomarkers in CRC, with reviews summarizing progress and clinical potential of methylation-based markers [11,12]. However, despite its biological and clinical relevance, the functional consequences of CIMP-related methylation changes and their impact on CRC progression and therapy resistance remain incompletely understood.

Integrative analysis of DNA methylation and transcriptomic data has become a widely adopted strategy for studying epigenetic regulation in cancer, supported by a growing body of statistical and computational methods for multi-omics data integration [13,14]. To elucidate the epigenetic mechanisms underlying CIMP and identify genes whose expression is regulated by methylation, computational approaches integrating multi-omics data are essential. Several tools have been developed for this purpose, including idiffomix [15], which applies joint mixture models to detect differential methylation and expression simultaneously; mixOmics (DIABLO) [16], which identifies predictive multi-omics signatures using supervised latent-variable models; MethylMix [17,18], which defines discrete methylation states to identify methylation-driven genes; ELMER [19], which links enhancer methylation to gene expression to reconstruct transcriptional regulatory networks; and MEAL [20], which provides region-level analyses of methylation–expression associations. These methods are generally model-based or network-oriented and often focus on predictive or latent representations of omics variation. In contrast, our approach adopts a transparent, promoter-centric framework that systematically tests biologically motivated thresholds and multiple promoter methylation summarization strategies through correlation- and regression-based analyses. This design prioritizes interpretability and cross-dataset robustness offering a simple means to evaluate methylation–expression relationships and identify candidate epigenetic biomarkers.

In this study, we investigated which computational strategy most robustly and interpretably identifies genes whose expression is regulated by promoter DNA methylation in CIMP-stratified colorectal cancer. We hypothesize that a promoter-centric regression framework, which models the combined effect of multiple CpG sites within gene promoters, provides a more biologically meaningful and transferable identification of epigenetically regulated genes. To this purpose, we developed and applied a computational strategy to identify genes whose expression is modulated by promoter methylation in a cohort of Colon Adenocarcinoma (COAD) patients from The Cancer Genome Atlas (TCGA) [21] identified according to CIMP status. We subsequently validated the best-performing method in other tumor types to assess its generalizability. By identifying novel epigenetic biomarkers, this approach enhances our understanding of CRC epigenetics and paves the way toward improved patient stratification and precision oncology.

## 2. Materials and Methods

### 2.1. Dataset Selection and CIMP-Based Stratification

This study was conducted using publicly available DNA methylation and gene expression data from The Cancer Genome Atlas (TCGA) [21]. Four cancer cohorts were analyzed: Colon Adenocarcinoma (COAD), Stomach Adenocarcinoma (STAD), Glioblastoma Multiforme (GBM), and Mesothelioma (MESO). These tumor types were selected because they have been reported to exhibit the CpG Island Methylator Phenotype (CIMP) [5]. The proposed method was developed and tested on the COAD dataset, which served as the primary cohort. The STAD, GBM, and MESO datasets were then used for validation to assess the method applicability across different tumor types.

The data has been retrieved from the v40.0 (29 March 2024) data release of NCI Genomic Data Commons (GDC), a data repository and computational platform for cancer researchers, through the TCGABiolinks R package (version 2.32.0) [22]. The DNA methylation data has been sequenced with the Infinium HumanMethylation450 BeadChip (Illumina Inc., San Diego, CA 92122 USA) [23], which covers about 450,000 CpG sites out of the approximately 28 millions in the human genome, and is provided in the form of beta values in range (0, 1), representing the methylation level of a CpG site. The transcriptome profiling data has been produced through RNA-seq and is provided in the form of raw counts, representing the expression levels of each gene.

This study exclusively considers primary tumor samples to ensure consistency across datasets. The patients have been stratified in three groups based on the CIMP status: CIMP-H, CIMP-L and Non-CIMP. The CIMP classification for the 4 datasets was obtained from the corresponding studies [24,25,26]. For the MESO dataset, the CIMP classification was not explicitly provided; however, a CIMP-index, a metric strongly correlated with CpG island methylation and computed at the CpG island level independently of gene-level annotations, was available. In the referenced study [27], the CIMP-index was calculated as the proportion of CpG islands per sample exhibiting a mean methylation beta value of at least 0.3, where island-level methylation was obtained by averaging beta values across all probes mapping to each CpG island. To achieve a balanced distribution of the three CIMP classes in our dataset, we classified samples with a CIMP-index of 0.5 or greater as CIMP-H, those with a CIMP-index of −0.5 or lower as Non-CIMP, and those with a CIMP-index between 0.5 and −0.5 as CIMP-L. Table 1 shows the number of primary tumor samples with both methylation and gene expression data, with the detail of CIMP groups stratification for the four datasets.

Analysis was restricted to CIMP-H and Non-CIMP groups due to their distinct methylation profiles, facilitating the identification of methylation-driven effects.

### 2.2. Data Preprocessing

Since our study focuses on the impact of promoter methylation on gene expression, we defined promoter regions as CpG sites included in the Illumina 450K manifest (Illumina Inc., San Diego, CA 92122 USA) that are located within 1500 base pairs upstream of a transcription start site (TSS1500). In addition, we included CpGs annotated as promoter-associated in the Illumina manifest. These annotations are based on genomic proximity to TSSs rather than direct experimental evidence of promoter activity, but they are likely to capture regions that influence transcription initiation. We note that this promoter definition may not capture all regulatory elements, such as distal enhancers or alternative promoters, and is limited by the coverage of the Illumina 450K array. To assess the robustness of our approach, the method was validated on independent cohorts (STAD, GBM, MESO), demonstrating consistent methylation-driven gene patterns across multiple tumor types.

To avoid confounding effects, CpGs located on sex chromosomes (X and Y) were excluded to mitigate sex-specific methylation biases, and CpGs overlapping common single nucleotide polymorphisms (SNPs) were removed, as these positions are prone to sequence variability that can affect probe binding. In addition, probes with missing values in at least one sample were excluded from the analysis to prevent bias and ensure the robustness of the statistical results.

The raw expression counts were normalized performing Trimmed Mean of M-values normalization (TMM) using the EdgeR [27] Bioconductor package (version 4.2.1) across all primary tumor samples, enabling accurate comparisons of gene expression between samples avoiding count biases caused by transcript length and sequencing depth.

Batch correction has not been applied since methylation and expression TCGA data are preprocessed and normalized to reduce technical variability.

### 2.3. Integrated Analysis of Differential Methylation and Gene Expression

An integrated differential methylation and differential gene expression analysis has been performed with the aim of identifying the genes that are both significantly differentially methylated and differentially expressed between the CIMP-H and Non-CIMP groups.

The differential methylation analysis was performed with the limma [28] package (version 3.60.4), while the differential gene expression analysis was conducted using EdgeR [27].

To investigate epigenetic regulation, it is essential to verify whether differentially methylated regions (DMRs) overlap with regulatory regions of differentially expressed (DE) genes. The methylation status of each gene promoter was approximated with the average beta value of the CpGs located within the relative region. For each gene, a promoter is considered differentially methylated based on both the Δβ (difference between beta values) or the log_2_ ratio and if the adjusted *p*-value is significant (lower than 0.05). In contrast, differential gene expression is determined solely based on the log_2_ fold changes and the adjusted *p*-value.

To select which genes exhibit a significant difference in either methylation or expression, between the two groups, several combinations of differential thresholds for the two omics were tested, as shown in Table A1. Thresholds used are based on those reported in other studies [29,30,31].

Finally, the selected thresholds were Δβ ≥ 0.2 for methylation and |log_2_FC| ≥ 1.3 for gene expression, as they represented a trade-off between retaining biologically relevant genes and excluding those with negligible methylation or expression differences between the two groups. The methylation threshold (Δβ ≥ 0.2) is widely accepted and used in previous studies [31,32]. The statistical significance of the differential expression threshold (|log_2_FC| ≥ 1.3) was validated through a sample size and power analysis, conducted with FPR = 0.05 and power = 0.9, using the RNASeqPower R package (version 1.44.0).

Based on these thresholds, the differentially methylated and expressed genes have been classified into five groups. Hypermethylated genes (Δβ ≥ 0.2) were either downregulated (log_2_FC ≤ −1.3) or upregulated (log_2_FC ≥ 1.3). Similarly, hypomethylated genes (Δβ ≤ −0.2) were downregulated (log_2_FC ≤ −1.3) or upregulated (log_2_FC ≥ 1.3). All other cases were considered not significant.

**Figure 1:**
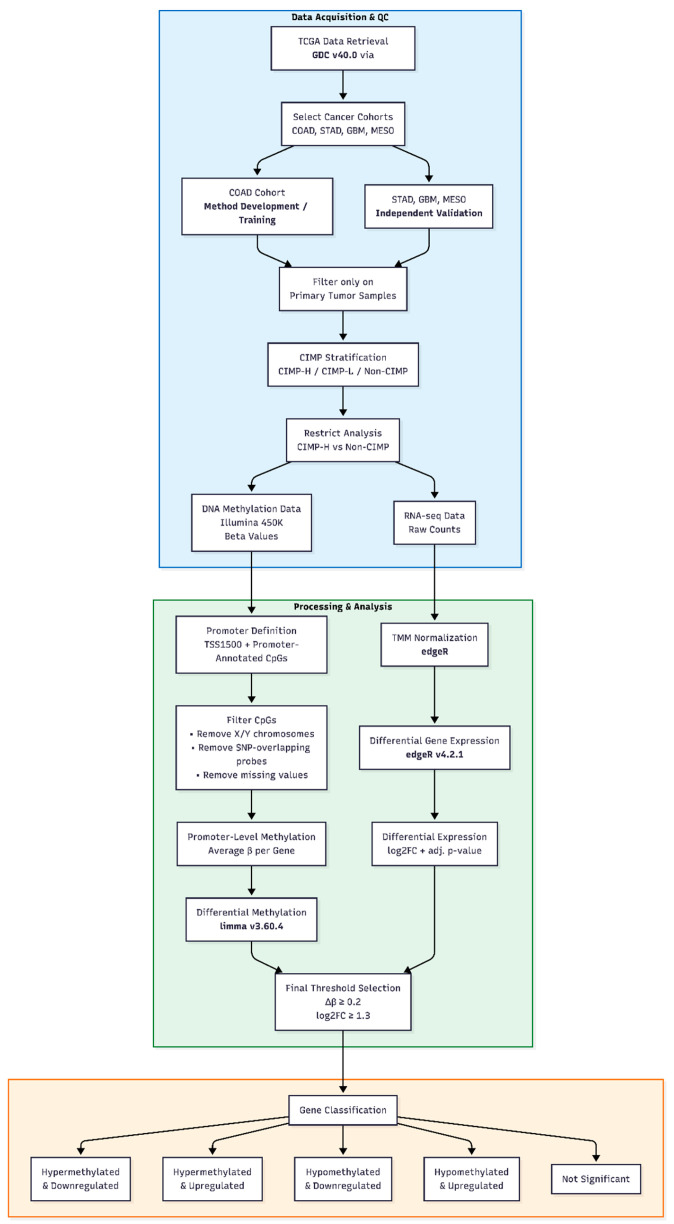
Overview of the analytical workflow for integrated DNA methylation and gene expression analysis.

Figure 1 provides a schematic overview of the complete analytical workflow, from data retrieval and preprocessing to integrated differential methylation and gene expression analysis.

Publicly available TCGA DNA methylation (Illumina HumanMethylation450) and RNA-seq data were retrieved and primary tumor samples from COAD, STAD, GBM, and MESO cohorts were selected. Samples were stratified according to CIMP status, and the analysis was restricted to CIMP-H and Non-CIMP groups. DNA methylation data were filtered and summarized at the promoter level, while RNA-seq data were normalized using TMM. Differential methylation and differential gene expression analyses were performed independently and subsequently integrated. Final gene selection was based on predefined methylation (Δβ ≥ 0.2) and expression (|log_2_FC| ≥ 1.3) thresholds, leading to the classification of genes into five methylation–expression categories. The method was developed using the COAD cohort and validated on STAD, GBM, and MESO datasets.

### 2.4. Methylation-Expression Correlation: Spearman- and Regression-Based Approaches

Identifying the genes that are both differentially expressed and methylated is a necessary, but not sufficient, condition for determining epigenetically regulated genes. It is also crucial to assess the relationship between promoter methylation and gene expression.

To this end, various approaches have been developed, leveraging either Spearman correlation or linear regression to assess the relationship between promoter methylation and gene expression.

Spearman correlation was chosen to measure the monotonic relationship between methylation and gene expression. The Spearman-based methods involve computing, for each gene and for all available CIMP-H and Non-CIMP samples, the Spearman correlation between a methylation-based metric and the corresponding gene expression. Then, only genes with a correlation greater than 0.4 and an adjusted *p*-value below 0.05 were considered, as done in a similar study [30]. The Regression-based methods involve fitting, for each gene and for all CIMP-H and Non-CIMP samples, a linear regression model with one or more methylation-based features as predictors and the gene expression level as the response variable. These models are not intended for predictive purposes, but rather to assess how well promoter methylation explains gene expression variability within our data. To quantify this relationship, we evaluated both the adjusted R^2^ and the overall statistical significance of each model. The adjusted R^2^ metric was used to measure the proportion of variance in gene expression explained by promoter methylation, while the overall model *p*-value, derived from the F-statistic (using pf(summary(lm_fit)$fstatistic [1], ...) in R (version 4.4.1)), was used to assess whether the observed association was statistically significant. An adjusted R^2^ value greater than 0.5 was interpreted as indicating that methylation explains a substantial portion of the variability in gene expression, provided that the model *p*-value was also statistically significant (*p* < 0.05).

**Figure 2:**
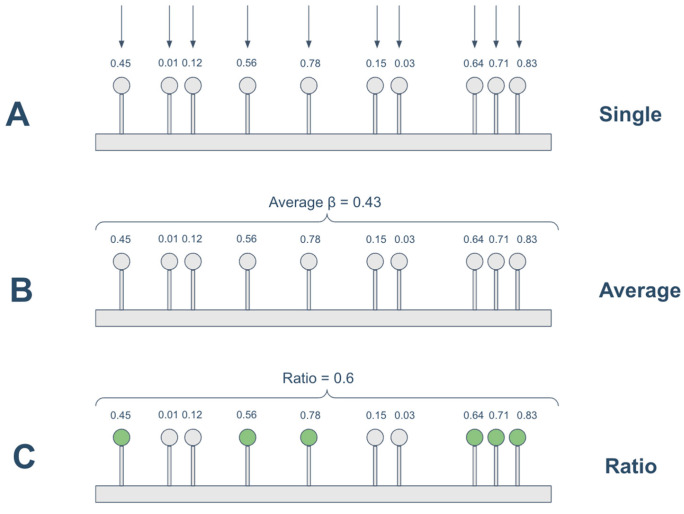
Strategies for handling methylation data. (**A**) single: the unit methylation data is the beta value of each individual CpG; (**B**) average: the promoter methylation status is calculated as the average of the beta values located on the promoter region; (**C**) ratio: the promoter methylation status is calculated as the ratio between methylated CpGs (β ≥ 0.3) over all CpGs located on the promoter.

Both the Regression-based and Spearman-based models were tested under three different approaches for handling methylation on the promoter, as shown in Figure 2 and outlined below:

• single: the unit methylation data is the beta value of each individual CpG site;
• average: the promoter methylation status is calculated as the average of the beta values located on the promoter region;
• ratio: the promoter methylation status was calculated as the ratio of methylated CpGs to the total number of CpGs in the promoter region. A CpG was considered methylated if its beta value was ≥0.3, following previous studies [26,33]. This threshold captures partially methylated CpGs that may influence transcription. More stringent cutoffs (e.g., β ≥ 0.5 or 0.7) could miss biologically relevant intermediate methylation, but future analyses may examine the impact of alternative thresholds.

Thus, for example, in the Spearman-single approach the beta value of each single CpG on the gene promoter is correlated with the expression of the gene, the correlations obtained are then averaged to have a single aggregated measure; while in the Spearman-average and Spearman-ratio, the expression of the gene is correlated with the average beta values of promoter CpGs and the ratio of methylated CpGs on the gene promoter, respectively.

Similarly, the Regression-single approach computes for each gene the adjusted R^2^ of a linear regression model having the gene expression as response variable and the beta values of all the CpGs on gene promoter as predictors; while the Regression-average and Regression-ratio consider as predictors the average beta value on gene promoter and the ratio of methylated CpGs on the promoter region, respectively.

Finally, for each method, the identified genes are ranked according to the corresponding score.

The different methods have been tested on the COAD dataset, and their results have been compared in terms of Spearman correlation coefficients or adjusted R^2^ scores to assess which methods are more effective at identifying high-scoring epigenetically regulated genes. For the three approaches within each of the two categories, the medians of the score distributions have been compared using a Kruskal-Wallis non-parametric test and we counted the number of genes for which each approach achieves the maximum score among the methods in the same category. Then, the overlapping between the list of genes identified with the different methods was considered and a qualitative evaluation was finally made based on the capability of the approaches to find relevant genes reported in other independent studies, such as *MLH1* [34].

To determine the most suitable method, we prioritized the one that identified a large number of genes, with significant overlap with those detected by other methods, which can be interpreted as a form of validation, and that exhibited biological relevance.

The best method was selected and validated on the STAD, GBM, and MESO datasets.

It is important to note that the proposed analyses are designed as an unsupervised discovery framework rather than a supervised predictive model. No ground-truth labels are available that definitively define whether a gene is epigenetically regulated by promoter methylation across all samples. Consequently, classical performance metrics such as sensitivity, specificity, positive predictive value, and negative predictive value cannot be computed. Instead, methodological robustness was assessed through comparative analysis across multiple correlation- and regression-based strategies, evaluation of internal concordance among methods, and biological plausibility of the identified genes.

### 2.5. Validation Against Independent Studies

To assess the consistency of our results, the identified genes for COAD and STAD were compared with the results reported in another study [24] from which the CIMP classification was obtained. For each gene, the TCGA samples in which the gene is epigenetically silenced are reported in the study. To compare our results, the percentages of epigenetically silenced samples for each gene in the CIMP-H and Non-CIMP groups were calculated. The difference between the two percentages was then used as a metric to assess differential epigenetic silencing between the two groups. This difference can be considered as a consistency metric against an independent definition of epigenetic silencing. The validation against independent studies was not intended to estimate predictive performance, but rather to assess consistency with an external, independently derived definition of epigenetic silencing. This strategy provides an indirect assessment of biological relevance and robustness in the absence of a gold-standard reference set.

As a further step, the identified genes were searched in the literature to confirm their association with cancer epigenetic mechanisms.

### 2.6. Code Availability

The scripts used for the analysis are available at the Zenodo repository https://doi.org/10.5281/zenodo.17416495.

## 3. Results

The Results section is structured to guide the reader from global epigenetic differences associated with CIMP status to the identification and validation of candidate genes whose expression is associated with promoter DNA methylation. First, we characterize differential methylation and gene expression patterns between CIMP-H and Non-CIMP tumors across datasets (Section 3.1), providing an overview of the epigenetic context considered in this study. We then systematically compare alternative methylation–expression integration strategies and motivate the selection of a Regression-based approach (Section 3.2). Using this approach, we identify and prioritize, within each tumor type, genes showing consistent associations between promoter methylation and expression (Section 3.3). Finally, we compare the resulting gene sets with those reported in an independent study based on an orthogonal definition of epigenetic silencing (Section 3.4), providing external support for the consistency of the proposed framework.

### 3.1. Comparative Analysis of Differential Methylation and Gene Expression Between CIMP-H and Non-CIMP Groups

**Figure 3:**
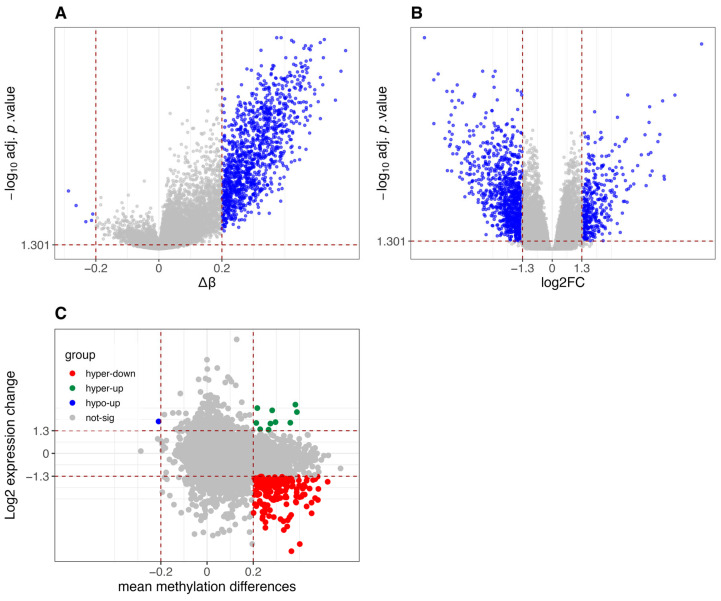
Differential methylation and gene expression analysis between CIMP-H and Non-CIMP groups in TCGA-COAD dataset. (**A**) Volcano plot of differentially methylated promoters. Red vertical lines indicate β-value thresholds of −0.2 and 0.2. The y-axis represents −log10 (adjusted *p*-values, FDR corrected using the Benjamini-Hochberg method), and promoters with adjusted *p*-value < 0.05 are considered significant. (**B**) Volcano plot of differentially expressed genes. Red vertical lines indicate log2 fold-change thresholds of −1.3 and 1.3. The y-axis represents −log10 (adjusted *p*-values, FDR corrected using Benjamini-Hochberg via limma/edgeR), and genes with adjusted *p*-value < 0.05 are considered significant. (**C**) Scatter plot integrating differential methylation and gene expression. Genes are classified into five categories: hypomethylated and upregulated (hypo-up), hypomethylated and downregulated (hypo-down), hypermethylated and downregulated (hyper-down), hypermethylated and upregulated (hyper-up), and not significant.

**Figure A1:**
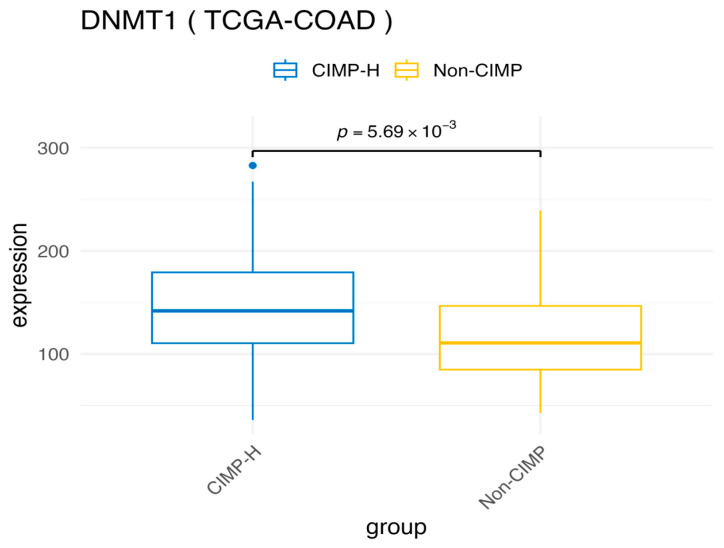
Comparison of DNMT1 expression between the CIMP-H and Non-CIMP groups.

As expected, CIMP-H samples in the COAD dataset exhibit global hypermethylation compared to Non-CIMP samples (Figure 3A), a hallmark of the CIMP molecular subtype. This is further corroborated by the increased expression of DNMT1, the key enzyme responsible for maintaining DNA methylation [35], in CIMP-H samples compared to Non-CIMP, (Figure A1). Elevated DNMT1 levels in CIMP-H tumors have been previously reported in CRCs, further supporting its role in sustaining aberrant DNA methylation patterns in these tumors [29].

Figure 3B shows the differential gene expression between the two groups, with a high number of significantly downregulated genes (956) in CIMP-H compared to Non-CIMP, versus upregulated ones (394).

Figure 3C reveals that, when integrating differential methylation and gene expression, a substantial group of genes are hypermethylated and downregulated in CIMP-H patients. This result is consistent with expectations and supports the assumption that hypermethylation is one of the key factors playing a silencing role in the regulation of gene expression.

**Figure A2:**
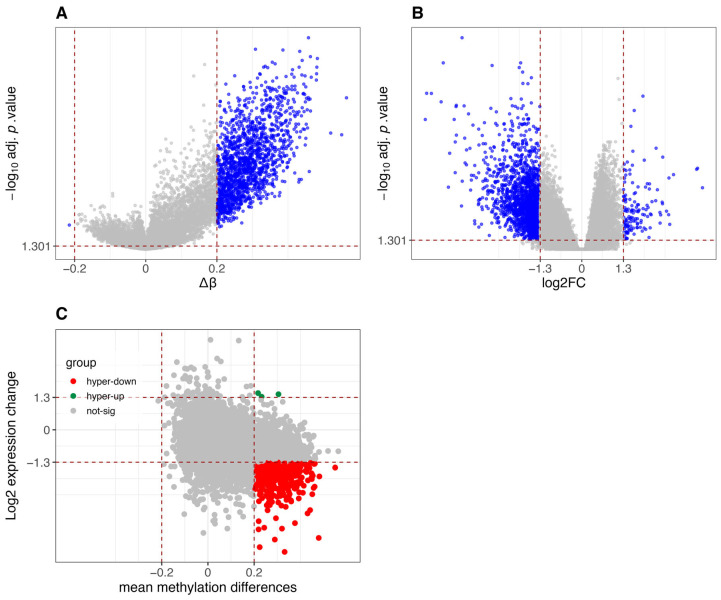
Differential methylation and gene expression analysis between CIMP-H and Non-CIMP groups in TCGA-STAD dataset. (A) Volcano plot of differentially methylated promoters. Red vertical lines indicate β-value thresholds of −0.2 and 0.2. The y-axis represents −log10 (adjusted *p*-values, FDR corrected using the Benjamini-Hochberg method), and promoters with adjusted *p*-value < 0.05 are considered significant. (B) Volcano plot of differentially expressed genes. Red vertical lines indicate log2 fold-change thresholds of −1.3 and 1.3. The y-axis represents −log10 (adjusted *p*-values, FDR corrected using Benjamini-Hochberg via limma/edgeR), and genes with adjusted *p*-value < 0.05 are considered significant. (C) Scatter plot integrating differential methylation and gene expression. Genes are classified into five categories: hypomethylated and upregulated (hypo-up), hypomethylated and downregulated (hypo-down), hypermethylated and downregulated (hyper-down), hypermethylated and upregulated (hyper-up), and not significant.

**Figure A3:**
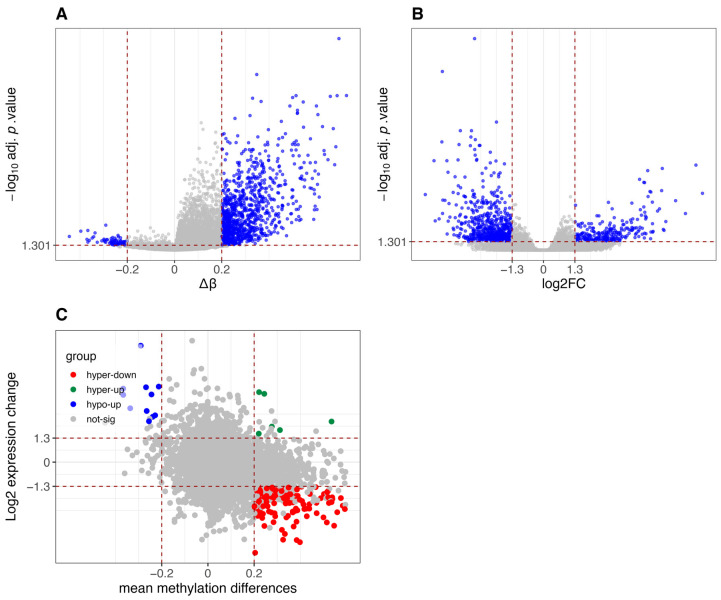
Differential methylation and gene expression analysis between CIMP-H and Non-CIMP groups in TCGA-GBM dataset. (A) Volcano plot of differentially methylated promoters. Red vertical lines indicate β-value thresholds of −0.2 and 0.2. The y-axis represents −log10 (adjusted *p*-values, FDR corrected using the Benjamini-Hochberg method), and promoters with adjusted *p*-value < 0.05 are considered significant. (B) Volcano plot of differentially expressed genes. Red vertical lines indicate log2 fold-change thresholds of −1.3 and 1.3. The y-axis represents −log10 (adjusted *p*-values, FDR corrected using Benjamini-Hochberg via limma/edgeR), and genes with adjusted *p*-value < 0.05 are considered significant. (C) Scatter plot integrating differential methylation and gene expression. Genes are classified into five categories: hypomethylated and upregulated (hypo-up), hypomethylated and downregulated (hypo-down), hypermethylated and downregulated (hyper-down), hypermethylated and upregulated (hyper-up), and not significant.

**Figure A4:**
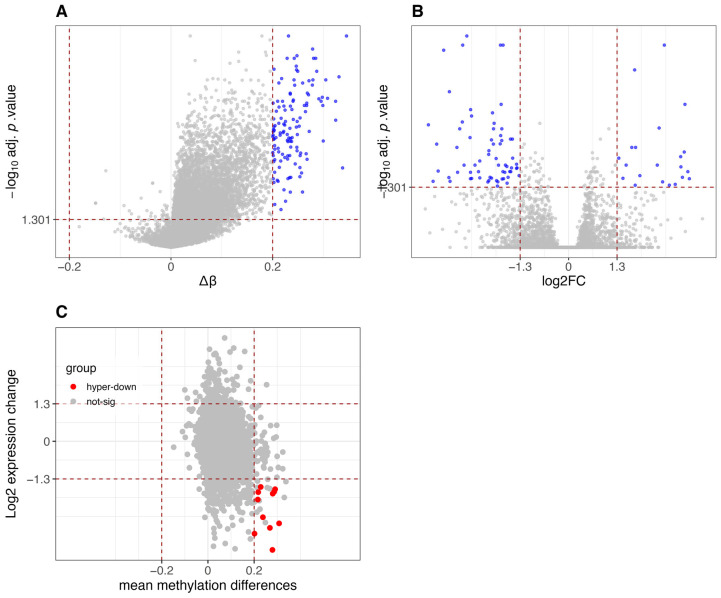
Differential methylation and gene expression analysis between CIMP-H and Non-CIMP groups in TCGA-MESO dataset. (A) Volcano plot of differentially methylated promoters. Red vertical lines indicate β-value thresholds of −0.2 and 0.2. The y-axis represents −log10 (adjusted *p*-values, FDR corrected using the Benjamini-Hochberg method), and promoters with adjusted *p*-value < 0.05 are considered significant. (B) Volcano plot of differentially expressed genes. Red vertical lines indicate log2 fold-change thresholds of −1.3 and 1.3. The y-axis represents −log10 (adjusted *p*-values, FDR corrected using Benjamini-Hochberg via limma/edgeR), and genes with adjusted *p*-value < 0.05 are considered significant. (C) Scatter plot integrating differential methylation and gene expression. Genes are classified into five categories: hypomethylated and upregulated (hypo-up), hypomethylated and downregulated (hypo-down), hypermethylated and downregulated (hyper-down), hypermethylated and upregulated (hyper-up), and not significant.

Similar results have been found for the differential analysis of the STAD, GBM and MESO datasets, as reported in Figure A2, Figure A3 and Figure A4, where CIMP-H samples show global hypermethylation compared to Non-CIMP samples and there is a substantial group of hypermethylated and downregulated genes.

### 3.2. Results of Methylation-Expression Correlation Methods and Method Selection

**Figure 4:**
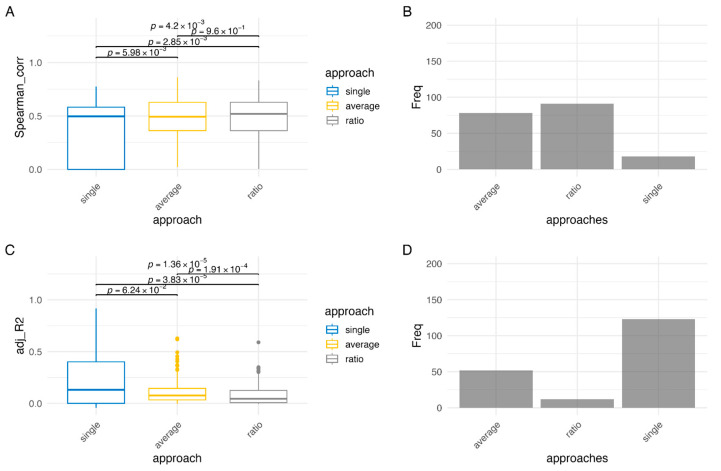
Comparison between the methods. (**A**) Comparison between distributions of Spearman correlation coefficients obtained with Spearman-based approaches (single, average, ratio). (**B**) Counts showing the number of genes for which each Spearman-based approach achieves the maximum score among the Spearman-based methods. (**C**) Comparison between distributions of adjusted R^2^ scores obtained with Regression-based approaches (single, average, ratio). (**D**) Counts showing the number of genes for which each Regression-based approach achieves the maximum score among the Regression-based methods.

We applied the Spearman-based and the Regression-based methods with the three metrics for methylation (single, average, ratio) on the COAD dataset. When comparing the scores obtained from the Spearman-based methods, there is no significant difference between the average and ratio approaches, with a 0.96 *p*-value on the Kruskal-Wallis test (Figure 4A). Despite this, the ratio approach yields the highest Spearman correlation score for most genes (48.7%), although the effect size is minimal. Figure 4B shows the number of genes for which each of the three approaches yields the maximum Spearman-based score. These counts represent an intermediate comparison metric used to evaluate the relative performance of the Spearman-based methods; they are not the criteria for selecting the final list of epigenetically regulated genes. As expected, the single approach results in low correlation coefficients for many genes, as it is unlikely that the methylation status of one single CpG can significantly impact gene expression levels. Notably, the Spearman-single metric represents the average correlation of individual CpGs for each gene, which can dilute strong effects from a single CpG. All three methods have a median score around 0.5 which suggests, since there is a group of genes having a much lower score, that for a subset of genes there is a significant correlation between promoter methylation and gene expression.

The distributions of adjusted R^2^ scores for Regression-based methods, in Figure 4C, shows that the average and ratio approaches lead to low adjusted R^2^ scores for most genes, with mean adj-R^2^ = 0.1119 and SD = 0.1156 for the average approach and mean adj-R^2^ = 0.0868 and SD = 0.1116 for the ratio approach. The only two genes associated with a score higher than 0.5 in both approaches are *PAX9* and *TTC9B*. On the other hand, the scores of the single approach, despite having a median (0.1322) that is not significantly higher than that of the average approach (0.0769), have an upper quartile with 39 genes exceeding 0.5 and 9 genes achieving scores above 0.8 (*MLH1*, *CHFR*, *TMEM176B*, *ZNF350*, *ZNF570*, *ZNF530*, *ZNF347*, *ZNF461*, *ZNF470*). Figure 4D confirms that the single approach yields the highest scores for more genes than the average or ratio approaches. The apparent discrepancy with the Spearman-single approach occurs because the Regression-single approach considers all CpGs on a gene’s promoter simultaneously as predictors, allowing the model to capture the combined effect of multiple CpGs or the strong effect of a single influential CpG, whereas the Spearman-single metric averages correlations across CpGs, potentially underestimating individual CpG contributions.

Notably, the *MLH1* gene, whose hypermethylation is often associated with the CIMP subtype in colorectal cancer [34], has the highest adjusted R^2^ of 0.916.

**Figure 5:**
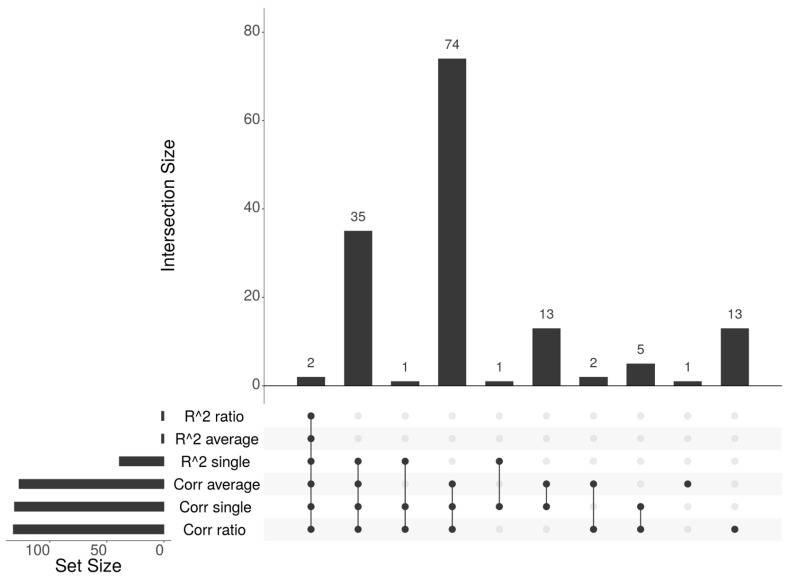
UpSet plot showing the overlaps among the gene lists identified by the methods.

There is a significant overlap between the list of genes identified by the different methods, as in all pairwise comparisons, the majority of genes in the smaller list are included in the larger one, as shown in the upSet plot in Figure 5. Specifically, the only two genes identified by the Regression-based average and ratio approaches (*PAX9* and *TTC9B*), are confirmed by all other methods. Moreover, 37 of the genes identified by the Regression-based single approach (39) are identified also by all the Spearman-based methods, except for *LARP6* that is not identified by the average approach and *GAL* that is not identified by average and ratio, while all 39 genes are identified by the Spearman-based single approach. Among the four methods able to identify a significant number of epigenetically regulated genes, the single Regression-based method has confirmed genes identified by different Spearman-based approaches. Additionally, this approach identified the *MLH1* gene, known for its biological association with the CIMP subtype and its role in cancer progression due to its DNA repair function [34], as the highest scoring gene. To summarize, this method identified a significant number of genes (39), with 95% overlap with those detected by other methods, and included biologically relevant genes, such as *MLH1*. Therefore, it was selected for validation on the STAD, GBM, and MESO datasets.

### 3.3. Identified Epigenetically Regulated Genes

The selected method was used to identify the epigenetically regulated genes in the datasets under study. The top 10 genes identified as significantly epigenetically regulated on the COAD dataset are reported in Table 2, while in Table 3, Table 4 and Table 5 the genes identified on the STAD, GBM and MESO datasets, respectively, are reported. The full list of genes are reported in Appendix A Table A2, Table A3, Table A4 and Table A5.

There is no overlap between the genes identified in the gastrointestinal datasets and those in the GBM and MESO datasets. However, 11 genes overlap between the 39 genes identified in the COAD dataset and the 23 genes identified in the STAD dataset. Among the common genes, the most important is *MLH1*, as mentioned earlier. An enrichment analysis on the common identified genes using the “KEGG 2021 Human” and “WikiPathways 2024 Human” ontologies reveals that the VANGL2 gene is associated with the Wnt signaling pathway [36], whose dysregulation has been linked to uncontrolled cell growth and tumor development.

While our study does not uncover new molecular mechanisms of DNA methylation itself, it highlights candidate genes whose epigenetic regulation in CIMP tumors has been underexplored. These genes may contribute to tumor progression and represent valuable starting points for future functional investigations.

### 3.4. Validation Against Independent Studies

**Figure 6:**
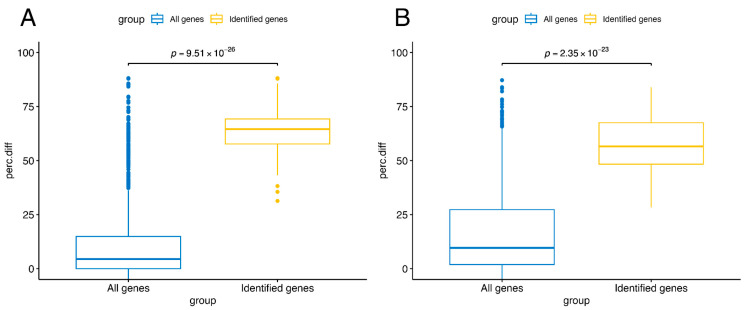
Validation of identified genes in TCGA-COAD and TCGA-STAD against epigenetically silenced from independent study. For each gene reported in the independent study by Liu, Y. et al. [25], the difference in the percentages of epigenetically silenced genes between CIMP-H and Non-CIMP groups was calculated according to their results. In that study, a gene is considered epigenetically silenced if more than half of its probes are epigenetically silenced, and a probe is defined as epigenetically silenced if, in at least 1% of tumor samples, it has a beta value ≥ 0.3 and the mean z-score of the methylated group is <−1.65 with FDR-corrected *p*-value < 0.001. Comparing the distributions of these percentage differences (perc.diff) between the identified genes (yellow) and the overall gene population (blue), both in COAD (**A**) and STAD (**B**), the identified genes are confirmed as epigenetically silenced by the other study. The difference in percentages of epigenetically silenced samples is not a performance metric, but a consistency metric against an independent definition of epigenetic silencing.

Figure 6 compares the distributions of the difference in the percentages of epigenetically silenced samples between the CIMP-H and Non-CIMP groups, calculated as described in the Methods section, in the overall gene population and in the genes identified by our approach. In the independent study [24], a gene is considered epigenetically silenced if more than half of its probes are epigenetically silenced; a probe is defined as epigenetically silenced if, in at least 1% of tumor samples, it has a beta value above 0.3 (classified as methylated) and the mean z-score of the methylated group is lower than −1.65 with an FDR-corrected *p*-value < 0.001. It can be observed that, for both COAD (Figure 6A) and STAD (Figure 6B), the genes identified by our approach exhibit a difference within the upper quartile of the overall gene population. This finding confirms that the identified genes are also reported as differentially epigenetically silenced in the results of this independent study.

As expected, *MLH1* was identified as the top epigenetically regulated gene in both gastrointestinal datasets, COAD and STAD. *MLH1* is a key player in DNA mismatch repair, and its promoter hypermethylation leads to transcriptional silencing, causing microsatellite instability (MSI). Our findings further support this link, as 69.8% of CIMP-H samples in COAD and 69.2% in STAD were classified as MSI-H, while the majority of Non-CIMP cases were microsatellite stable (MSS) (Table A6 and Table A7).

**Figure A5:**
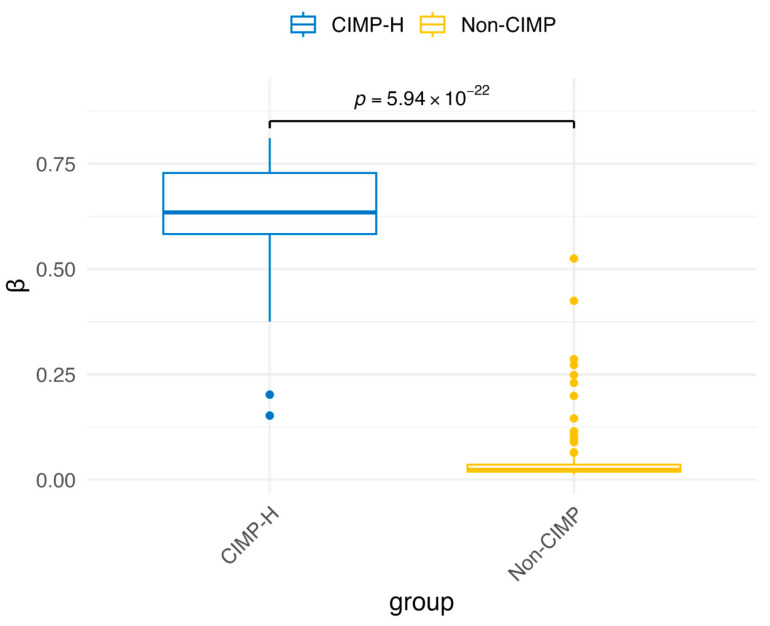
Comparison of β values distributions on cg13601799 between the CIMP-H and Non-CIMP groups.

Another gene whose methylation is associated with CIMP in colorectal cancer is *CDKN2A* [37], a tumor suppressor. This gene was not identified in our study, since it is not differentially expressed in our data (log_2_FC = 0.09), although it is differentially methylated (Δβ = 0.26). Moreover, in the study [24] used for CIMP classification, its methylation status is evaluated solely based on the probe cg13601799. As shown in Figure A5, there is significant difference in methylation for this CpG site in our colon cancer data as well. Therefore, our findings align with the expected differential methylation of *CDKN2A*, but it was not identified in our study due to its lack of differential expression.

## 4. Discussion

Aberrant promoter DNA methylation modulates the expression of genes controlling processes such as DNA repair, cell-cycle regulation, and signaling pathways implicated in cancer progression. Therefore, computationally analyzing multiomic datasets to identify genes that are epigenetically regulated in a cohort of cancer patients has the potential to prioritize candidate diagnostic biomarkers and epigenetic therapeutic targets, providing a hypothesis-generating resource for downstream functional studies.

Our developed computational approach confirmed a significant correlation between promoter methylation and gene expression for a subset of genes. After testing different alternative approaches, which included Spearman correlation, linear regression, and various strategies for handling methylation data, the best model for capturing this correlation was the one based on the adjusted R^2^ coefficients, using the methylation beta values of its promoter CpGs as predictors. However, we note that correlation does not establish causality: promoter methylation–expression associations can arise from confounding factors such as copy-number variation, tumor purity, cell-type composition, co-methylated genomic domains, batch effects, and broader transcriptional programs. As a result, some candidates may represent false positives. Future extensions that incorporate partial correlations or multivariable models (e.g., controlling for CNV, purity, and mutation burden), replication across cohorts, and orthogonal functional evidence (e.g., chromatin accessibility or targeted perturbation) will be essential to strengthen causal inference.

In principle, the proposed method may be generalized on other cancer datasets to identify genes that are epigenetically regulated by DNA methylation between distinct subgroups, such as the CIMP-H and Non-CIMP subtypes. Moreover, cross-dataset comparison of the identified gene lists reveals tumor-type–specific epigenetic regulation and highlights shared versus context-dependent methylation–expression relationships. In particular, we noted a significant overlap between the genes identified in the COAD and STAD datasets, suggesting a similar epigenetic regulation profile between these two gastrointestinal tumors, while there is no overlap with the genes identified in the GBM and MESO datasets, suggesting that epigenetic regulation may affect different genes and pathways across different tumor types.

To assess the functional relevance of the identified genes in cancer, we investigated their known biological roles and associations with tumor progression. Given the exploratory and computational nature of the study, this interpretation is intended to contextualize the findings within established colorectal cancer biology rather than to infer novel mechanisms. Notably, a subset of genes identified in both the COAD and STAD datasets is functionally linked to key cancer-related processes, including DNA mismatch repair, mitotic checkpoint regulation, and Wnt signaling. These findings, detailed below, are consistent with previous studies and further reinforce the robustness of our analytical approach.

Among these, *MLH1*, a key player in the mismatch repair system, was epigenetically silenced in 14% of COAD and 19.7% of STAD samples. Similarly, *CHFR*, a tumor suppressor involved in the maintenance of the mitotic checkpoint, showed silencing frequencies of 32% in COAD and 29.3% in STAD. *EPM2AIP1*, which encodes for a protein interacting with the phosphatase laforin, although less characterized, was also found to be silenced in 9.9% of COAD and 14% of STAD samples [38], suggesting a potential, yet underexplored, role in gastrointestinal tumorigenesis.

We also identified genes related to the Wnt signaling pathway, including *VANGL2* and *FUZ*. The latter has already been described in the literature for its prognostic value in various cancers, including STAD, where its overexpression correlates with poor overall survival [39]. This highlights FUZ as an epigenetically regulated gene that may affect tumor growth and malignancy through the Wnt signaling pathway. In addition, *PCDHGC3*, a member of the protocadherin gene cluster, was found hypermethylated in our dataset. Promoter hypermethylation of *PCDHGC3* has been proposed as a potential biomarker for gastrointestinal neuroendocrine carcinomas and is frequently altered in COAD and STAD [40,41].

Additionally, many of the identified genes for the COAD and STAD datasets belong to the Zinc Finger (ZNF) family, whereas no ZNF genes were identified in the GBM and MESO datasets. The role of the Zinc Finger Proteins in the regulation of gastrointestinal tumors has been reported and explored in another study [42], although further research is needed to elucidate their specific involvement. Alterations in ZNF gene methylation have also been observed in Barrett’s esophagus, where they show predictive potential for progression to esophageal adenocarcinoma [43]. These observations suggest that the epigenetic deregulation of ZNF genes may contribute to gastrointestinal tumorigenesis and warrant further investigation.

In the TCGA-GBM dataset, several genes of potential biological and clinical relevance were also identified. For instance, *VILL*, which encodes for a member of the villin/gelsolin family, showed altered methylation in 1p/19q-deleted gliomas, and its upregulation has been associated with poor prognosis [44]. *FBXO17*, a gene involved in cell cycle regulation, has been correlated with unfavorable survival outcomes in high-grade gliomas [45,46]. Likewise, *EMP3*, which encodes a transmembrane epithelial protein and has been previously reported as a prognostic biomarker in glioblastoma, is linked to reduced proliferation and migration when silenced [47].

Other notable genes include *KHNYN*, a cofactor of the zinc finger ZAP protein, whose expression inversely correlates with overall survival; *TUBA1C*, which encodes the tubulin alpha 1c protein, associated with poor prognosis in low-grade gliomas; and ZDHHC12, a zinc finger protein associated with glioma growth and malignancy [48,49,50].

Although *TSTD1* (thiosulfate sulfurtransferase-like domain containing 1) has not been directly studied in glioblastoma, it is of interest due to its epigenetic deregulation in other cancers. In particular, hypomethylation of *TSTD1* has been associated with altered treatment response in breast cancer [51], suggesting a broader relevance in tumor biology.

Importantly, the primary contribution of this work is not the discovery of novel methylation mechanisms, but the systematic prioritization of genes whose promoter methylation correlates with expression in CIMP-stratified tumors. Many of these genes have been underexplored and may provide a foundation for future studies investigating their functional roles in tumor progression.

Altogether, these findings emphasize the biological plausibility of our approach and its capacity to identify functionally relevant genes across multiple tumor types. The enrichment of known cancer-related genes and pathways, especially in COAD and STAD, supports the validity of the computational pipeline and provides a valuable foundation for future experimental validation and functional studies.

Despite its promising results, the accuracy and significance of this exploratory analysis in identifying epigenetically regulated genes in cancer are mainly limited by the size of the datasets, the constraints of the technologies used to obtain the data, and the data-driven, unsupervised nature of the method proposed. The limited number of TCGA patients and the imbalance between CIMP-H and Non-CIMP cases may undermine the significance of the results. In particular, the results obtained on the GBM datasets are less robust compared to the others, since the GBM dataset is smaller and includes only 3 CIMP-H samples. A similar concern applies to MESO, where the number of CIMP-H cases is very low, further limiting statistical power. Accordingly, conclusions regarding CIMP-dependent methylation in GBM and MESO should be considered exploratory and interpreted with caution. Moreover, the methylation data available for the TCGA datasets were obtained using the HumanMethylation450 BeadChip, which covers a small fraction of the CpG sites in the human genome compared to other emerging technologies characterized by a higher coverage, such as the MethylationEPIC v2.0 array, or covering the whole genome, such as Whole Genome Bisulfite Sequencing (WGBS) and long-reads Oxford Nanopore sequencing (ONT). As a result, some relevant contributions to the overall epigenetic regulation may be missed. More significant results could be achieved by using larger, more balanced datasets and methylation data sequenced with techniques that provide better coverage than the 450 k.

A limitation of this study is that potential batch effects and covariates, such as sex, were not explicitly modeled in the differential and correlation analyses. Although TCGA data are preprocessed and normalized to reduce technical variability, unaccounted confounders could still contribute to noise in methylation or expression measurements. In particular, batch effects or biological covariates such as sex, age, tumor stage, tumor purity, and cell-type composition could inflate or attenuate the observed promoter methylation–expression associations for certain genes, potentially generating false positives or false negatives. Given that our focus was on significant differences between CIMP-H and Non-CIMP tumors, these effects are unlikely to alter the main findings, but future extensions should include covariate-adjusted models or batch correction methods to ensure robustness across larger and more heterogeneous cohorts.

A potential concern when introducing new computational approaches is the absence of classical performance metrics and explicit positive or negative controls. In our study, this limitation reflects the unsupervised nature of the problem: there is currently no comprehensive gold-standard set of genes that can be unambiguously labeled as epigenetically regulated or non-regulated by promoter methylation across tumor types. Well-characterized examples such as MLH1 represent only a small and biased subset and cannot serve as a complete reference for supervised evaluation. As a result, sensitivity, specificity, or predictive accuracy cannot be meaningfully estimated.

To address this, we adopted a multi-layered validation strategy based on internal concordance across methods, recovery of well-established biologically relevant genes, reproducibility across independent tumor cohorts, and consistency with an external study defining epigenetic silencing using independent criteria. Together, these analyses provide complementary evidence supporting the robustness and biological relevance of the identified genes, while avoiding overinterpretation of the method as a predictive classifier. Accordingly, the proposed framework should be interpreted as a hypothesis-generating tool for epigenetic discovery rather than a diagnostic or predictive model.

Moreover, a potential shortcoming of the selected Regression-based single method is that it involves the comparison of the R^2^ values of linear models having a different number of predictors, since the number of CpG sites on the promoter varies across genes. To address this, the adjusted R^2^ metric has been considered instead of the R^2^, in order to avoid bias towards larger models and ensure a higher degree of robustness in the method.

## 5. Conclusions

In this study, we presented a computational approach to identify genes epigenetically regulated by DNA methylation across different cancer types. By modeling the relationship between promoter methylation and gene expression using adjusted R^2^ metrics, we were able to effectively capture biologically meaningful associations and prioritize genes potentially involved in tumor progression. The method was first developed and tested on a COAD patient cohort stratified by CIMP status and subsequently validated on STAD, GBM, and MESO datasets, demonstrating its robustness and potential for generalization.

Our results highlight consistent patterns of epigenetic regulation within gastrointestinal tumors, including the silencing of *MLH1*, *CHFR*, and other genes linked to DNA repair, cell cycle control, and Wnt signaling. We also identified tumor-specific epigenetic signatures in GBM and MESO, further supporting the biological relevance of the findings.

Despite these promising results, limitations remain. These include the relatively small and imbalanced patient cohorts, particularly in the GBM dataset, potential unaccounted batch effects and the restricted CpG site coverage of the HumanMethylation450 array. The unsupervised nature of the method also constrains the ability to quantitatively compare alternative approaches. Nonetheless, the use of adjusted R^2^ mitigates model complexity bias and enhances methodological robustness.

The resulting ranked gene lists and validated analytical framework provide concrete resources for downstream functional studies and for the evaluation of methylation-based biomarkers in CIMP-associated cancers. Overall, our work underscores the value of computational frameworks in epigenetic research and demonstrates their potential to uncover novel biomarkers and therapeutic targets. Further development of the method is needed to address its limitations and take advantage of more comprehensive datasets and advanced sequencing technologies. Refining the method holds significant potential for deepening our understanding of epigenetic regulation and its clinical applications in cancer. Although not intended for immediate clinical deployment, this framework could have clinical relevance by refining patient stratification through identification of epigenetically distinct tumor subgroups, highlighting candidate methylation-based biomarkers (e.g., *MLH1*, *CHFR*, and *ZNF* genes) for diagnostic or prognostic purposes, and prioritizing genes for functional validation in epigenetic therapy studies, such as targeted demethylation or CRISPR/dCas9-mediated epigenetic editing.

## Figures and Tables

**Table 1:** Number of primary tumor samples, classified as CIMP-H, CIMP-L and Non-CIMP with both methylation and gene expression data in the selected TCGA datasets.

Dataset	Primary Tumors	CIMP-H	CIMP-L	Non-CIMP
TCGA-COAD	292	43	116	102
TCGA-STAD	373	52	63	197
TCGA-GBM	60	3	0	48
TCGA-MESO	87	23	24	26

**Table 2:** Top 10 genes identified on the TCGA-COAD dataset.

Gene	adj-R^2^	Model *p*.Value	Meth Δβ	Meth adj.*p*.Value	Expr |log_2_FC|	Expr adj.*p*.Value
*MLH1*	0.9160	9.757 × 10^−51^	0.2696	1.005 × 10^−18^	−1.6650	4.159 × 10^−21^
*CHFR*	0.9155	8.039 × 10^−60^	0.2692	4.361 × 10^−17^	−1.3277	9.634 × 10^−14^
*TMEM176B*	0.8590	3.332 × 10^−54^	0.2144	2.907 × 10^−26^	−1.4168	4.478 × 10^−16^
*ZNF350*	0.8545	4.427 × 10^−60^	0.2320	1.861 × 10^−18^	−1.3189	4.834 × 10^−13^
*ZNF570*	0.8445	1.232 × 10^−50^	0.2716	5.133 × 10^−32^	−1.7217	9.649 × 10^−20^
*ZNF530*	0.8400	5.514 × 10^−52^	0.2465	1.043 × 10^−21^	−2.0575	9.957 × 10^−20^
*ZNF347*	0.8114	5.530 × 10^−52^	0.2658	4.999 × 10^−17^	−1.4471	9.699 × 10^−12^
*ZNF461*	0.8051	3.875 × 10^−46^	0.3696	4.018 × 10^−39^	−1.5938	7.787 × 10^−18^
*ZNF470*	0.8011	1.548 × 10^−45^	0.3167	5.709 × 10^−31^	−2.5464	1.020 × 10^−27^
*ZNF665*	0.7960	5.481 × 10^−47^	0.2725	2.512 × 10^−13^	−1.6427	7.221 × 10^−10^

**Table 3:** Top 10 genes identified on the TCGA-STAD dataset.

Gene	adj-R^2^	Model *p*.Value	Meth Δβ	Meth adj.*p*.Value	Expr |log_2_FC|	Expr adj.*p*.Value
*MLH1*	0.8625	5.111 × 10^−82^	0.3309	1.735 × 10^−38^	−1.3131	2.522 × 10^−21^
*SPAG16*	0.8108	7.904 × 10^−82^	0.2190	1.693 × 10^−22^	−1.3352	6.162 × 10^−9^
*ZNF549*	0.7659	2.865 × 10^−73^	0.2224	1.639 × 10^−18^	−1.3527	2.983 × 10^−11^
*ZNF530*	0.7521	9.503 × 10^−69^	0.2278	5.345 × 10^−34^	−1.3645	2.023 × 10^−11^
*EPM2AIP1*	0.7326	1.265 × 10^−59^	0.3128	5.515 × 10^−39^	−1.5758	1.698 × 10^−25^
*FUZ*	0.7183	1.696 × 10^−59^	0.2771	1.017 × 10^−32^	−1.8069	8.646 × 10^−19^
*PCDHGC3*	0.6902	5.196 × 10^−58^	0.2714	9.279 × 10^−34^	−1.4244	2.100 × 10^−1^
*ZNF415*	0.6898	6.833 × 10^−63^	0.2125	6.708 × 10^−24^	−1.3567	7.768 × 10^−10^
*ZNF518B*	0.6557	1.171 × 10^−50^	0.2012	7.820 × 10^−20^	−1.5725	1.583 × 10^−13^
*TTC9B*	0.6534	6.150 × 10^−53^	0.2325	2.510 × 10^−30^	1.3267	5.290 × 10^−13^

**Table 4:** Top 10 genes identified on the TCGA-GBM dataset.

Gene	adj-R^2^	Model *p*.Value	Meth Δβ	Meth adj.*p*.Value	Expr |log_2_FC|	Expr adj.*p*.Value
*VILL*	0.9262	2.472 × 10^−17^	0.3397	6.303 × 10^−17^	−1.5257	2.000 × 10^−4^
*FAM50B*	0.9094	1.020 × 10^−12^	0.2101	9.718 × 10^−8^	−1.8676	4.300 × 10^−3^
*TRIP4*	0.9025	2.564 × 10^−22^	0.3142	4.801 × 10^−20^	−1.6195	3.990 × 10^−14^
*FBXO17*	0.8961	1.733 × 10^−22^	0.3254	5.730 × 10^−26^	−4.1905	1.744 × 10^−28^
*EMP3*	0.8679	2.235 × 10^−19^	0.3466	2.387 × 10^−24^	−2.8986	2.188 × 10^−9^
*FCHSD1*	0.8678	2.272 × 10^−19^	0.2259	1.471 × 10^−6^	−1.5234	4.983 × 10^−6^
*KHNYN*	0.8342	7.694 × 10^−15^	0.5379	6.496 × 10^−23^	−1.8945	4.784 × 10^−9^
*TUBA1C*	0.8262	1.959 × 10^−13^	0.2737	1.811 × 10^−23^	−1.8564	4.579 × 10^−7^
*ZDHHC12*	0.8093	7.718 × 10^−16^	0.2317	2.483 × 10^−7^	−1.4266	4.424 × 10^−5^
*TSTD1*	0.7886	7.635 × 10^−12^	0.3710	3.538 × 10^−6^	−3.4774	4.892 × 10^−6^

**Table 5:** Top 10 genes identified on the TCGA-MESO dataset.

Gene	adj-R^2^	Model *p*.Value	Meth Δβ	Meth adj.*p*.Value	Expr |log_2_FC|	Expr adj.*p*.Value
*NMNAT3*	0.7119	6.622 × 10^−12^	0.2379	3.593 × 10^−7^	−2.6290	1.200 × 10^−3^
*TMEM220*	0.6577	8.033 × 10^−11^	0.2176	1.281 × 10^−8^	−1.7578	4.226 × 10^−5^
*RNF208*	0.5908	7.443 × 10^−8^	0.2284	1.447 × 10^−9^	−1.577	2.290 × 10^−2^
*OCIAD2*	0.5642	1.103 × 10^−6^	0.2867	5.852 × 10^−9^	−1.744	3.270 × 10^−2^
*CMBL*	0.5576	2.265 × 10^−8^	0.2682	6.128 × 10^−8^	−2.9973	6.900 × 10^−3^

## Data Availability

The R script used for the analysis is available at the “R script to identify epigenetically regulated genes in multi-omic cancer datasets” Zenodo repository https://doi.org/10.5281/zenodo.17416495. The code is written in R (version 4.4.1) and is Platform independent. The code is distributed under the Creative Commons Attribution 4.0 International license. No new data were generated in this study, as all analyses were conducted using publicly available cancer datasets from the TCGA portal. As such, no additional data are available beyond those existing public resources.

## Abbreviations

The following abbreviations are used in this manuscript:

CRC	Colorectal Cancer
CIMP	CpG Island Methylator Phenotype
COAD	Colon Adenocarcinoma
DE	Differentially Expressed
GBM	Glioblastoma Multiforme
GDC	NCI Genomic Data Commons
MESO	Mesothelioma
SNPs	Single Nucleotide Polymorphisms
STAD	Stomach Adenocarcinoma
TCGA	The Cancer Genome Atlas
TSS	Transcriptional Start Site

## Appendix A

**Table A1:** Combinations of differential methylation and expression thresholds.

Diff Methylation Threshold	Diff Expression Threshold (log_2_FC)
log_2_FC ≥ log_2_(1.05) or log_2_FC ≤ log_2_(0.95)	|log_2_FC| ≥ 1
	|log_2_FC| ≥ 1.3
	|log_2_FC| ≥ 1.5 |log_2_FC| ≥ 2
|log_2_FC| ≥ 1.2	|log_2_FC| ≥ 1 |log_2_FC| ≥ 1.3 |log_2_FC| ≥ 1.5
	|log_2_FC| ≥ 2
|∆β| ≥ 0.1	|log_2_FC| ≥ 1 |log_2_FC| ≥ 1.3 |log_2_FC| ≥ 1.5
	|log_2_FC| ≥ 2
|∆β| ≥ 0.2	|log_2_FC| ≥ 1 |log_2_FC| ≥ 1.3 |log_2_FC| ≥ 1.5
	|log_2_FC| ≥ 2
|∆β| ≥ 0.3	|log_2_FC| ≥ 1 |log_2_FC| ≥ 1.3 |log_2_FC| ≥ 1.5 |log_2_FC| ≥ 2

**Table A2:** Genes identified in TCGA-COAD.

Genes	adj-R^2^	Model *p*.Value	Meth Δβ	Expr |log_2_FC|
*MLH1*	0.9160142218	9.7573301802 × 10^−51^	0.2696545	−1.665074
*CHFR*	0.9155066294	8.0396914024 × 10^−60^	0.269226246	−1.32773189
*TMEM176B*	0.8590336994	3.3328801090 × 10^−54^	0.214482	−1.416893
*ZNF350*	0.8545988847	4.4278195945 × 10^−60^	0.2320397	−1.318949
*ZNF570*	0.8445531222	1.2327774024 × 10^−50^	0.271608	−1.721735
*ZNF530*	0.8400372136	5.5140546279 × 10^−52^	0.2465791	−2.057586
*ZNF347*	0.8114975965	5.5301184056 × 10^−52^	0.2658497	−1.447183
*ZNF461*	0.8051404749	3.8759977094 × 10^−46^	0.3696641	−1.593841
*ZNF470*	0.8011371961	1.5487570193 × 10^−45^	0.3167758	−2.546431
*ZNF665*	0.7960067914	5.4817053359 × 10^−47^	0.2725474	−1.64277
*FUZ*	0.7777392954	2.5091023605 × 10^−40^	0.2435446	−1.825009
*NHLRC1*	0.7712743506	4.0644414518 × 10^−40^	0.3041523	−2.612487
*ZNF518B*	0.7698917405	6.0750372604 × 10^−40^	0.2561586	−2.0496
*ZSCAN18*	0.7674092534	1.9739502642 × 10^−38^	0.2257022	−1.548055
*RAB32*	0.7542588235	4.8471220810 × 10^−38^	0.273973	−1.748984
*EPM2AIP1*	0.7421457065	2.0466792632 × 10^−32^	0.3108179	−1.668124
*ZNF790*	0.7166895081	2.2100181493 × 10^−33^	0.3014525	−1.802083
*GSTM3*	0.7049150426	3.8140509799 × 10^−35^	0.2129139	−1.305608
*PCDHGC3*	0.7017538942	5.1118072493 × 10^−33^	0.2672849	−1.390975
*LARP6*	0.6748827652	2.9597049278 × 10^−34^	0.3334754	−1.758083
*BBS5*	0.6656853753	4.8494375654 × 10^−32^	0.2283376	−1.345157
*GNG4*	0.6557856995	5.0993101991 × 10^−26^	0.3651545	−5.601045
*PAX9*	0.6531760303	6.1897381747 × 10^−31^	0.3822773	2.796667
*ZNF287*	0.648190007	9.0622211349 × 10^−29^	0.3056667	−1.320021
*MYEF2*	0.6400660127	3.2151261728 × 10^−29^	0.4526309	−3.425723
*ZNF345*	0.6273470281	4.2866553672 × 10^−27^	0.3128804	−1.834955
*SCRN1*	0.6178338195	1.9406897476 × 10^−27^	0.2349142	−1.310881
*TTC9B*	0.6120338869	7.9436566855 × 10^−29^	0.3603467	1.759013
*ZNF256*	0.608153717	3.8447277982 × 10^−25^	0.2595887	−1.940782
*DNM3*	0.6032257626	8.6872374039 × 10^−26^	0.2889181	−1.577103
*VANGL2*	0.5803117683	1.1544112232 × 10^−24^	0.2977	−2.432072
*TMEM176A*	0.5797805563	4.1558335632 × 10^−24^	0.251388	−1.813204
*KLF7*	0.5782472007	1.6506221588 × 10^−23^	0.2988387	−1.478293
*STC2*	0.5533759868	6.4931066518 × 10^−24^	0.3305407	−1.505171
*GAL*	0.5355766068	3.4449879148 × 10^−22^	0.2289686815	−2.82493938
*TRMT12*	0.5256366293	2.6472947155 × 10^−23^	0.2417141	−1.468805
*ACSL6*	0.5238291627	5.0859492373 × 10^−20^	0.3321547	−4.38583
*ADAM32*	0.5231521371	1.6271688295 × 10^−22^	0.3019306	−2.247536
*DENND2C*	0.5085058292	1.4737898361 × 10^−17^	0.2506021	−1.629142

**Table A3:** Genes identified in TCGA-STAD.

Genes	adj-R^2^	Model *p*.Value	Meth Δβ	Expr |log_2_FC|
*MLH1*	0.8625194402	5.1115944567 × 10^−82^	0.3309603	−1.313101
*SPAG16*	0.8108002593	7.9047197457 × 10^−82^	0.2190329	−1.335292
*ZNF549*	0.7659061963	2.8651608949 × 10^−73^	0.2224513	−1.352792
*ZNF530*	0.7521410701	9.5033608340 × 10^−69^	0.2278235	−1.364519
*EPM2AIP1*	0.7326966892	1.2653387700 × 10^−59^	0.3128086	−1.575874
*FUZ*	0.7183519805	1.6965220867 × 10^−59^	0.2771977	−1.806915
*PCDHGC3*	0.690208358	5.1966558535 × 10^−58^	0.271416	−1.424485
*ZNF415*	0.6898570341	6.8339031726 × 10^−63^	0.2125039	−1.356719
*ZNF518B*	0.6557446401	1.1716878367 × 10^−50^	0.2012544	−1.572526
*TTC9B*	0.6534714454	6.1507217211 × 10^−53^	0.2325077	1.326743
*STOX2*	0.6262812014	9.8970559076 × 10^−50^	0.3389031	−2.172084
*PPP1R9A*	0.6216226523	1.7206102252 × 10^−46^	0.2289913	−2.537084
*PYGO1*	0.6045898697	4.5360754050 × 10^−49^	0.21845	−1.49573
*ZNF512B*	0.5981651401	2.0369709415 × 10^−47^	0.2650061	−1.691932
*TUB*	0.5694345938	1.5470821262 × 10^−40^	0.2091746743	−1.72417821
*VANGL2*	0.5676130373	3.6114328641 × 10^−42^	0.3398634	−2.803349
*LARP6*	0.5645034498	1.7903179607 × 10^−42^	0.2753438	−1.619616
*NUP210*	0.5544244614	1.2961432358 × 10^−40^	0.23946828	−1.7079703
*ZSCAN18*	0.5467536543	2.3698137358 × 10^−36^	0.2450219	−1.674229
*NHLRC1*	0.5453508891	3.1074756177 × 10^−37^	0.262541	−2.077767
*PAIP2B*	0.534069801	5.2728482658 × 10^−36^	0.2455338	−1.795764
*ZNF300*	0.5334687427	1.4050379317 × 10^−39^	0.2397008	−1.31001
*CABYR*	0.5072583955	8.0060346562 × 10^−35^	0.2526839	−1.592365

**Table A4:** Genes identified in TCGA-GBM.

Genes	adj-R^2^	Model *p*.Value	Meth Δβ	Expr |log_2_FC|
*VILL*	0.9262673517	2.4725768581 × 10^−17^	0.3397819	−1.525788
*FAM50B*	0.9094371166	1.0201226544 × 10^−12^	0.2101957	−1.867682
*TRIP4*	0.9025516512	2.5642870743 × 10^−22^	0.3142913	−1.61957
*FBXO17*	0.8961476435	1.7337920747 × 10^−22^	0.3254043	−4.190574
*EMP3*	0.8679583888	2.2357782662 × 10^−19^	0.3466201	−2.898656
*FCHSD1*	0.8678618202	2.2724422625 × 10^−19^	0.2259016	−1.523468
*KHNYN*	0.8342158227	7.6944683535 × 10^−15^	0.5379234	−1.894583
*TUBA1C*	0.8262239337	1.9594887247 × 10^−13^	0.2737355	−1.856427
*ZDHHC12*	0.8093786572	7.7184376089 × 10^−16^	0.2317068	−1.426678
*TSTD1*	0.7886016084	7.6354783070 × 10^−12^	0.3710361	−3.477444
*RAB34*	0.7870253404	3.6418158146 × 10^−13^	0.367253	−2.712978
*XKR8*	0.7818698663	1.3668922651 × 10^−11^	0.3775083	−2.632459
*MARVELD1*	0.7664193197	6.8408254916 × 10^−14^	0.4048824	−1.322334
*KCNB1*	0.7546708328	6.3690196282 × 10^−12^	0.5351048	2.197043
*TOM1L1*	0.7536948059	6.8998771422 × 10^−12^	0.5146205	−3.254208
*CLIC1*	0.7321082475	6.8432347298 × 10^−9^	0.3569989	−1.734158
*LRRC61*	0.7236492858	9.4667143224 × 10^−8^	0.2163285	−2.899631
*ALDH7A1*	0.7176096928	3.3559684485 × 10^−13^	0.339439	−2.984188
*B3GNT5*	0.7117808817	6.0406915694 × 10^−11^	0.5755067	−1.936565
*PPCS*	0.7024967074	1.1598064690 × 10^−10^	0.2597928	−1.438017
*FABP5*	0.700038623	4.7278840357 × 10^−9^	0.2886457	−2.75996
*TTC12*	0.6936032628	5.4736520843 × 10^−10^	0.5353078	−2.154691
*PYROXD2*	0.6820653714	1.8500042218 × 10^−11^	0.2564855	−1.939967
*MIR155HG*	0.6767940524	8.3669349604 × 10^−11^	0.407702	−2.100055
*B3GNT7*	0.6724731901	4.8141644041 × 10^−9^	0.2946536	−1.592759
*ECHDC2*	0.670169979	4.2148141764 × 10^−11^	0.4173006	−2.530786
*EID3*	0.6485458465	3.4989560072 × 10^−9^	0.592721	−2.531831
*OSMR*	0.641828216	8.1542530517 × 10^−11^	0.2419406	−1.97559
*RBP1*	0.6248659311	2.1109179887 × 10^−9^	0.3841675936	−4.21122843
*FERMT1*	0.6111895937	1.6592323584 × 10^−9^	−0.3364869	2.915307
*LRRC34*	0.6099437671	6.3478581723 × 10^−8^	0.2528083	−1.948628
*TCEA3*	0.5991307379	1.0867475374 × 10^−9^	0.5596619	−2.702318
*STEAP3*	0.5970572542	9.8376572230 × 10^−9^	0.3717444	−2.009174
*CBLN3*	0.5849646858	2.1146152728 × 10^−7^	0.5835179	−2.35043
*NIPAL2*	0.5834731801	2.0029059943 × 10^−8^	0.2036887	−1.565651
*PDLIM4*	0.5379376026	1.6548089798 × 10^−6^	0.3202394	−3.657253
*ZIC5*	0.5184717764	4.3845145105 × 10^−7^	0.2867333	−2.217452
*CMYA5*	0.5093551602	5.1501871268 × 10^−6^	0.6127386	−2.663765
*CD109*	0.5007389969	1.6346569625 × 10^−7^	0.3198041	−1.770341

**Table A5:** Genes identified in TCGA-MESO.

Genes	adj-R^2^	Model *p*.Value	Meth Δβ	Expr |log_2_FC|
*NMNAT3*	0.71194155	6.6224383300 × 10^−12^	0.2379173	−2.629047
*TMEM220*	0.65779601	8.0332932199 × 10^−11^	0.2176805	−1.757845
*RNF208*	0.59085732	7.4437667235 × 10^−8^	0.2284904	−1.577897
*OCIAD2*	0.56425239	1.1039000636 × 10^−6^	0.2867297	−1.744734
*CMBL*	0.55763349	2.2651777226 × 10^−8^	0.268212	−2.997392

**Table A6:** Association between CIMP subgroups and MSI subgroups in the TCGA-COAD dataset.

	MSI-H	MSI-L	MSS	NA
CIMP-H	30	4	9	0
Non-CIMP	9	14	78	1

**Table A7:** Association between CIMP subgroups and MSI subgroups in the TCGA-STAD dataset.

	MSI-H	MSI-L	MSS
CIMP-H	36	7	9
Non-CIMP	9	20	168
